# Persistent order due to transiently enhanced nesting in an electronically excited charge density wave

**DOI:** 10.1038/ncomms10459

**Published:** 2016-01-25

**Authors:** L. Rettig, R. Cortés, J.-H. Chu, I. R. Fisher, F. Schmitt, R. G. Moore, Z.-X. Shen, P. S. Kirchmann, M. Wolf, U. Bovensiepen

**Affiliations:** 1 Fakultät für Physik, Universität Duisburg-Essen, Lotharstr. 1, D-47057 Duisburg, Germany; 2 Fachbereich Physik, Freie Universität Berlin, Arnimallee 14, D-14195 Berlin, Germany; 3 Abteilung Physikalische Chemie, Fritz-Haber-Institut der MPG, Faradayweg 4-6, D-14195 Berlin, Germany; 4 Geballe Laboratory for Advanced Materials, Department of Applied Physics, Via Pueblo Mall, Stanford, California 94305, USA; 5 SLAC National Accelerator Laboratory, Stanford Institute for Material and Energy Sciences, Menlo Park, 94025 California, USA

## Abstract

Non-equilibrium conditions may lead to novel properties of materials with broken symmetry ground states not accessible in equilibrium as vividly demonstrated by non-linearly driven mid-infrared active phonon excitation. Potential energy surfaces of electronically excited states also allow to direct nuclear motion, but relaxation of the excess energy typically excites fluctuations leading to a reduced or even vanishing order parameter as characterized by an electronic energy gap. Here, using femtosecond time- and angle-resolved photoemission spectroscopy, we demonstrate a tendency towards transient stabilization of a charge density wave after near-infrared excitation, counteracting the suppression of order in the non-equilibrium state. Analysis of the dynamic electronic structure reveals a remaining energy gap in a highly excited transient state. Our observation can be explained by a competition between fluctuations in the electronically excited state, which tend to reduce order, and transiently enhanced Fermi surface nesting stabilizing the order.

## Abstract (web summary)

Whilst excited electronic states may exhibit unique non-equilibrium behavior, order is inhibited by fluctuations. Here, the authors use femtosecond photoemission spectroscopy to demonstrate transient stabilization of charge density wave order in rare earth tritellurides after near-infrared excitation.

## Graphical abstract

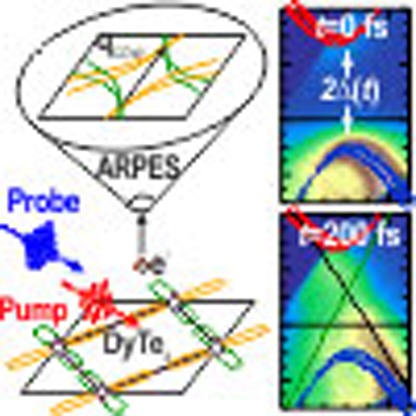

Ordered states in condensed matter emerge in thermodynamic equilibrium below a critical temperature *T*_c_ if competing thermal fluctuations are weak compared with the energy gain driving the order. This delicate balance depends critically on the dimensionality of a system, where low-dimensional structures introduce instabilities towards broken symmetry ground states, but on the same footing enhance fluctuations. Charge density waves (CDWs) form due to cooperative effects of electrons and the crystal lattice and represent widely studied model systems of such broken symmetry ground states. Under equilibrium conditions CDW formation can be described phenomenologically by effective mean field models^1^, but on a microscopic level a description is far from trivial, because the electronic and lattice contributions can hardly be disentangled due to their intrinsic coupling^2^,^3^. Optical excitation of CDW systems into non-equilibrium states faster than the characteristic time scales of electrons or phonons breaks this link and has opened opportunities to investigate the interactions underlying CDW formation^4^,^5^,^6^,^7^,^8^,^9^,^10^,^11^. In addition, tailored excitations of specific resonances in the mid-infrared have been reported to lead to novel, non-equilibrium material properties that are not accessible in thermal equilibrium^12^,^13^,^14^,^15^,^16^.

The formation of a CDW is driven by an instability of the electronic system to a spatially periodic perturbation. Particularly in quasi one-dimensional systems parallel parts of the Fermi surface (FS) are nested by an ordering vector ***q***_CDW_, and lead to a divergence of the electronic Lindhard susceptibility^1^. Electron-phonon (e-ph) coupling imprints this ordering tendency on the lattice and freezes a soft phonon mode into a periodic lattice distortion, leading to a complex many-body problem. While this is a widely considered explanation for CDW formation^1^,^17^, momentum-dependent e-ph coupling may modify this picture^3^,^18^,^19^. This coupled charge and lattice periodicity 1/*q*_CDW_ creates an energy gap 2Δ in the electronic structure due to Bragg scattering at the modulated charge density. Thereby the periodic lattice distortion and the CDW are stabilized by the strength of e-ph coupling and the diverging susceptibility, that is, the FS topology. Optical excitation creates fluctuations of the electron density, which on average reduce the electronic charge modulation and 2Δ. Simultaneously, coherent lattice dynamics may drive the system towards a high-symmetry state and decrease the lattice distortion, further reducing the CDW modulation and 2Δ. Such ultrafast suppression in 2Δ has been widely observed in previous experiments employing femtosecond time- and angle-resolved photoemission spectroscopy (trARPES) after femtosecond laser excitation^5^,^6^,^20^ in agreement with the theory^21^, which emphasizes the impact of optically induced fluctuations on the interaction leading to CDW formation. However, an enhanced driving force for CDW formation maintaining order in the non-equilibrium state was so far not observed.

Here we demonstrate a hindered melting of the CDW state after femtosecond laser excitation in the CDW model system RTe_3_ (R=Dy, Ho) through a residual gap 2Δ in a transient optically excited state. This residual 2Δ is fluence independent and is a consequence of improved FS nesting conditions in the excited state, for which we provide evidence through a complete analysis of 2Δ as a function of two independent momentum directions *k*_*x*_, *k*_*z*_, binding energy *E* and pump-probe delay *t*. This improved nesting increases the interactions underlying CDW formation which compete with incoherent fluctuations induced by the excitation. This surprising observation suggests a pathway to control material properties under non-equilibrium conditions relevant for a wide range of material classes with broken symmetry ground states.

## Results

### Static FS of RTe_3_

**Figure 1:**
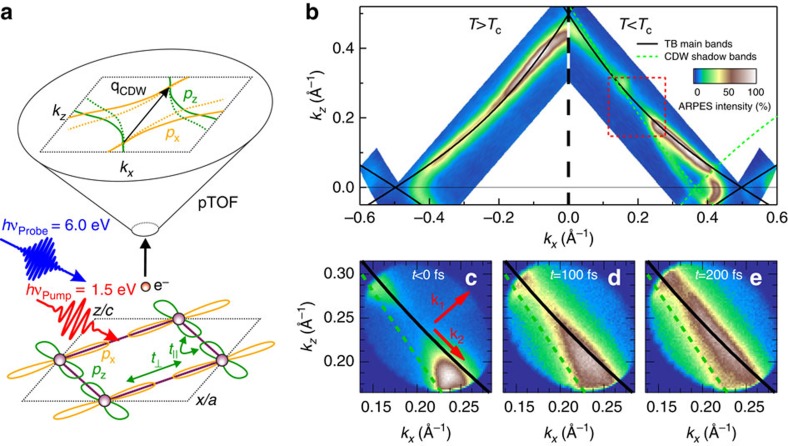
Time-dependent Fermi surface map. (**a**) Experimental scheme and TB model. The Te-5*p*_*x*/*z*_ orbitals in the Te-layers of RTe_3_ (bottom) are coupled by *t*_||_ and *t*_⊥_ describing interactions parallel and perpendicular to the Te chains and lead to a diamond-shaped Fermi surface in reciprocal space, which we excess by our position-sensitive time-of-flight photoelectron spectrometer (pTOF) (top). Nesting leads to shadow bands translated along **q**_CDW_ (dashed lines). (**b**) Static FS of HoTe_3_ for *T*=300 K>*T*_c_ (left) and *T*=180 K<*T*_c_ (right). Main and shadow TB bands are indicated by solid and dashed lines, respectively. In the CDW phase, energy gaps open on the Fermi surface where main and shadow bands overlap. The red square indicates the region investigated in panels (**c**–**e**). (**c**–**e**) Time-dependent FS of DyTe_3_ for various pump-probe delays at *T*<*T*_c_. The gapped FS transforms within 200 fs into a continuous, metallic state. Arrows in **c** indicate directions perpendicular (*k*_1_) and parallel (*k*_2_) to the FS, respectively. The weak intensity in the upper left corner in **c** stems from a partially twinned crystal domain.

We begin by analyzing the electronic structure of HoTe_3_ in thermal equilibrium using high-resolution laser-ARPES at *hν*=7 eV, which is representative for the series of rare-earth tritellurides. Its quasi-2D electronic structure is determined by square nets of Te-planes, which are stacked between buckled RTe layers that allow to tune the CDW temperature and gap size through chemical pressure^22^. Figure 1b (left) shows the continuous, metallic FS of HoTe_3_ for *T*>*T*_c_, which agrees well with tight binding (TB) calculations (solid lines) of the Te planes taking into account the overlap of Te-5*p*_*x*_ and 5*p*_*z*_ orbitals (refs 17, 18), for details see Supplementary Note 1 and Supplementary Fig. 1. The curvature of the diamond-shaped FS in the (*k*_*x*_, *k*_*z*_)-plane is determined by the ratio of orbital overlap along and perpendicular to the chains of *p*_*x*_/*p*_*z*_-orbitals in the TB model described by *t*_⊥_/*t*_||_ (ref. 17), see Fig. 1a. For *T*<*T*_c_ (Fig. 1b, right) CDW formation results in shadow bands (green dashed lines), which are translated along *k*_*z*_ by the nesting vector **q**_CDW_≈0.7c* (ref. 17) (Fig. 1a), and a CDW energy gap 2Δ opens in the vicinity of (*k*_*x*_, *k*_*z*_)=(0.15, 0.30) Å^−**1**^ where the FS is nested due to overlapping main and shadow bands (see Supplementary Fig. 1). With decreasing *k*_*z*_ the nesting gradually weakens and residual metallic pockets appear on the FS, where spectral weight is transferred into the shadow bands. Note that the imperfect nesting results in a *k*-dependent shift of the gap centre along the FS, while the full gap value 2Δ remains constant (see Supplementary Fig. 1). In the heavier members of RTe_3_, starting from TbTe_3_, also a second perpendicular CDW transition occurs along a* at a lower *T* (refs 18, 22). Here we concentrate on the first CDW transition with the larger 2Δ, which is well separated on the FS.

### Transient FS and band structure

Using femtosecond trARPES, see Fig. 1a, we investigate the electronic structure after fs laser excitation in DyTe_3_, which is very similar to HoTe_3_ (refs 17, 18). Figure 1c–e shows the gapped FS region (red box in Fig. 1b) probed by *hν*_probe_=6.0 eV after optical excitation with *hν*_pump_=1.5 eV pump pulses at an absorbed fluence of *F*=0.27 mJ cm^−2^ for selected time delays. A movie of the transient FS is also available (Supplementary Movie 1). We observe filling of the gapped region starting from the metallic pocket. At 200 fs a nearly ungapped FS is found, which is accompanied by a shift of spectral weight back to the TB main band. This provides direct evidence for an optically driven gap-closing transition. At a first glance the transient state at 200 fs is very similar to the situation at *T*>*T*_c_ and consistent with the TB model^6^,^23^.

**Figure 2:**
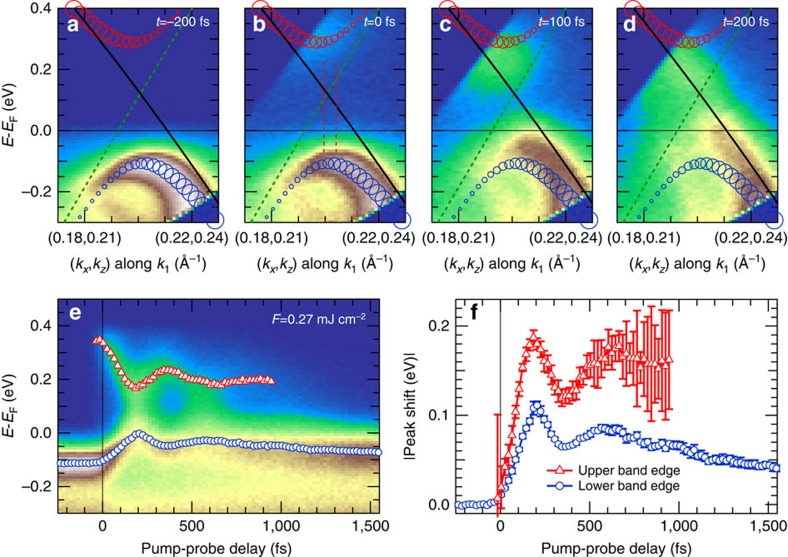
Time-dependent CDW band structure. (**a**–**d**) trARPES data along *k*_1_ for various pump-probe delays. Main and shadow TB bands are shown as solid and dashed lines, and symbols show lower (blue) and upper (red) band dispersion of the TB model including CDW gap, where the symbol sizes represent the spectral weight. (**e**) Spectra integrated around the region marked in **b** as a function of pump-probe delay. Red and blue symbols are peak positions of upper and lower CDW bands, respectively, obtained from a fitting procedure. (**f**) Peak shift of upper and lower band. Shown is the absolute value of the shift. Error bars represent 95% confidence intervals of the fits.

Our electron time-of-flight spectrometer^24^ allows extracting spectra along arbitrary in-plane momenta and we first analyze the trARPES intensity *I*(*E*, *k*_1_, *t*) with *k*_1_ perpendicular to the FS contour, see Fig. 1c and Supplementary Movie 2. Figure 2 shows an occupied band with CDW gap before excitation. At *t*=0 fs the unoccupied band at the top of the CDW gap is populated, which enables a direct determination of the full transient 2Δ. Both bands and the gap energy agree well with the interacting TB model (see Supplementary Note 1). Simultaneously, the occupied band is depleted, which implies hole excitation. With increasing pump-probe delay *t* the lower and upper bands move towards each other as the CDW order recedes and 2Δ is reduced. At *t*=100 fs the lower band reaches *E*_F_ and at *t*=200 fs, *E*(*k*_1_) is well described by the TB main band in agreement with the FS analysis above.

In Fig. 2e we plot trARPES spectra at **k**_F_ and clearly observe periodic variations of 2Δ. The peak positions of the lower and upper CDW bands are determined by Lorentzian line fits of *I*(*E*, *t*), for details see Supplementary Note 2 and Supplementary Fig. 2. Before excitation, the lower CDW band occurs at *E*−*E*_F_=−0.12(1) eV (blue markers). Beginning at *t*=0 fs, we monitor the upper CDW band at *E*−*E*_F_=0.34(3) eV (red markers) and determine 2Δ=0.46(4) eV from the energy difference of the upper and lower CDW peaks. With increasing *t* both lower and upper CDW bands shift towards *E*_F_. At *t*=200 fs the lower (upper) band reaches its maximum (minimum) and 2Δ is smallest before the bands shift away from *E*_F_ again at larger delays. Remarkably, two distinct peaks are observed in the spectra at all times (Supplementary Fig. 2), providing direct evidence for 2Δ remaining finite in the transient metallic state.

**Figure 3:**
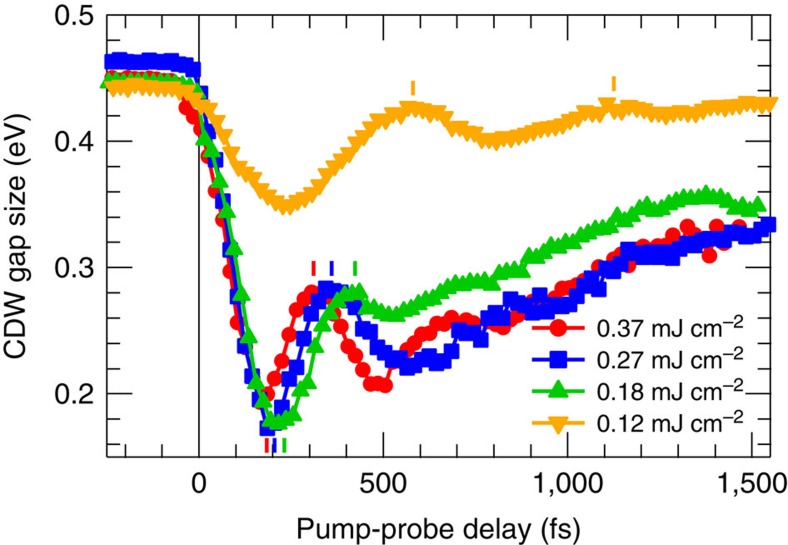
Time-dependent CDW gap size. Time-dependent CDW gap size 2Δ determined from the separation of upper and lower CDW band as a function of pump-probe delay for various fluences.

Figure 3 shows 2Δ(*t*) (see Supplementary Note 3 and Supplementary Fig. 3) for various fluences *F*. For *F*≤0.12 mJ cm^−2^ a relatively small decrease is followed by an oscillation with a period of ∼0.5 ps. This coherent response in a weakly perturbative regime lasts for several picoseconds and corresponds to the excitation of the CDW amplitude mode^1^,^6^,^23^,^25^,^26^,^27^,^28^. For higher fluence in a strongly perturbative regime *F*≥0.18 mJ cm^−2^, corresponding to ∼220 meV per unit cell (see Supplementary Note 4), which is comparable to the electronic energy gain upon CDW formation *δE*∼250 meV per unit cell estimated from the TB model (see Supplementary Note 1 and Supplementary Fig. 1), we find a stronger initial decrease of 2Δ, followed by a recovery of 2Δ overlaid by anharmonic and strongly damped oscillations. These oscillations are a fingerprint of the coherent atomic rearrangements during the collapse and recovery of the CDW order in the transient potential energy surface. The minimal value of 2Δ saturates at 170 meV independent of fluence for *F*>0.12 mJ cm^−2^. This previously unobserved residual 2Δ in the transient metallic state demonstrates the persistence of electronic order in a highly perturbed system, in contrast to equilibrium conditions at *T*>*T*_c_ (ref. 18). This incomplete suppression of the CDW state is an unexpected result because at the highest fluence an excess energy of almost twice the energy gained by gapping the electronic structure is injected and still the gap persists (Fig. 3).

### Asymmetric gap suppression

**Figure 4:**
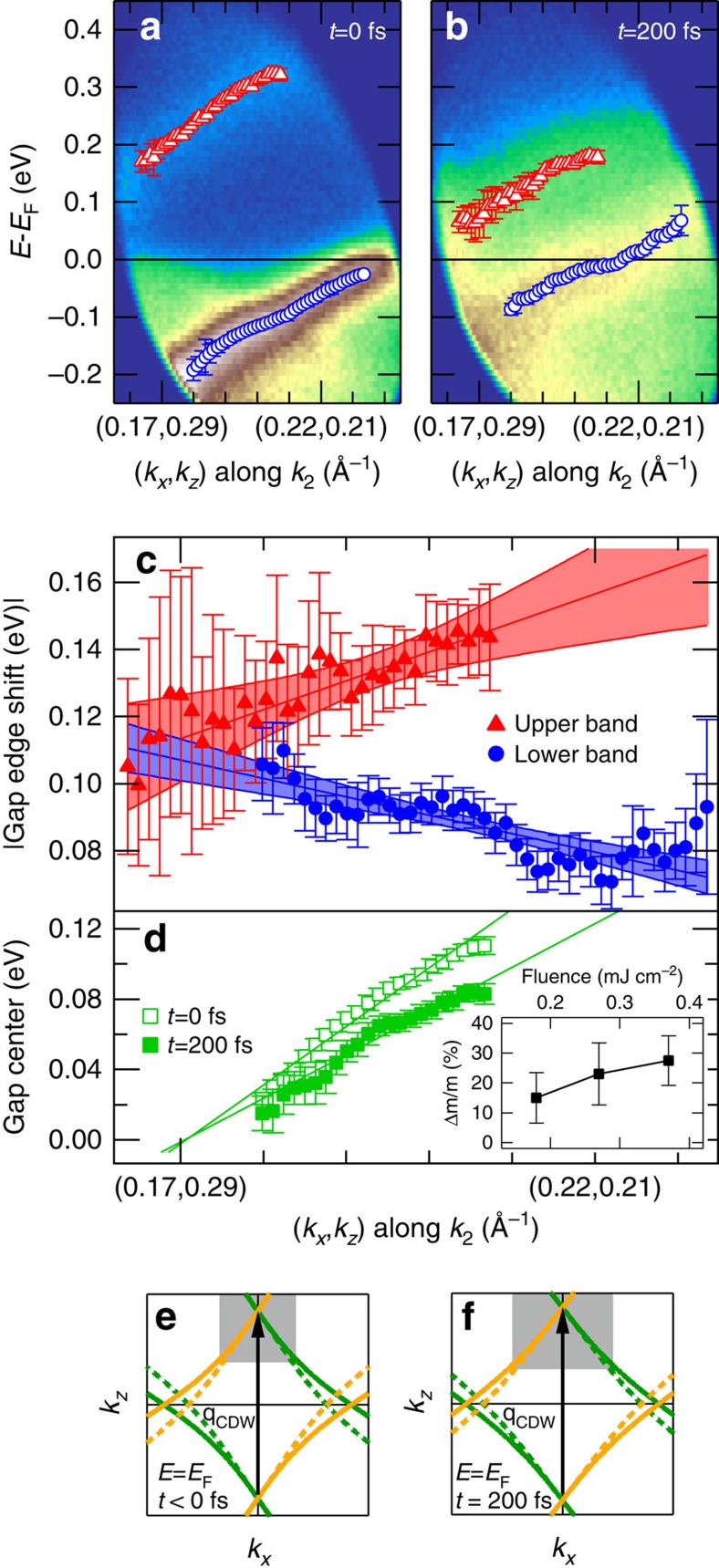
Gap collapse along the Fermi surface. (**a**,**b**) Electron dispersion along *k*_2_ for *t*=0 fs and *t*=200 fs, respectively. Markers denote energy positions of upper (red) and lower (blue) CDW bands. (**c**) Peak shift between *t*=0 fs and =200fs of upper and lower CDW band as function of position on the FS determined by fits, with error bars as 95% confidence intervals. Lines and shaded areas are linear fits and 95% confidence bands, respectively. (**d**) Centre of the CDW gap as a function of position on the FS at *t*=0 fs (open symbols) and at *t*=200 fs (filled symbols). Solid lines are linear fits. The inset shows the relative change of slope Δ*m*/*m* as function of fluence. (**e**,**f**) Tight binding bands for *t*_⊥_=0.35 eV (**e**) and *t*_⊥_=0.25 eV (**f**). The grey areas marks the regions of good nesting where main (solid) and shadow (dashed) band lines overlap.

Our experimental approach further allows for the analysis of the symmetry of 2Δ. A closer look at the transient peak positions in Fig. 2e reveals a larger pump-induced shift of the upper than of the lower CDW peak, indicating an asymmetric decrease of 2Δ. From the relative shift of both bands in Fig. 2f we find a maximal shift of 110 meV for the lower band (blue), while the upper band (red) changes by 60% more up to 190 meV. The momentum dependence of *I*(*E*, *k*_2_, *t*) along *k*_2_ (see Fig. 1c) parallel to the FS contour is shown in Figs 4a,b for *t*=0 and 200 fs and exhibits lower and upper CDW bands dispersing along *k*_2_. A full movie is available as Supplementary Movie 3. This relates to a shift of the centre of 2Δ along the FS because 2Δ(**k**) is only centred around *E*_F_ for perfect nesting^17^ near (*k*_*x*_, *k*_*z*_)=(0.17, 0.29) Å^−1^. To study the transient state, it is helpful to look at the pump-induced changes where the gap is minimal (*t*=200 fs). The pump-induced peak shifts (see Supplementary Note 5 and Supplementary Fig. 4) |*E*(*k*_2_)^up,down^(200 fs)−*E*(*k*_2_)^up,down^(0 fs)| are depicted in Fig. 4c and exhibit a pronounced dependence on the FS position: With decreasing *k*_*x*_, the transient shift of the upper band decreases, while the shift of the lower band increases, leading to a smaller asymmetry of the gap reduction. Finally, at (*k*_*x*_, *k*_*z*_)∼(0.17, 0.29) Å^−1^ both peak shifts become equal and the CDW gap decreases symmetrically around its centre. These observations manifest in a transient reduction of the dispersion of the gap centre with *k*_2_, as seen in Fig. 4d.

## Discussion

Such a transient change of the gap centre indicates an ultrafast modification of the nesting condition. The nesting vector and the curvature of the bands (Fig. 1) determine the band crossing of main and shadow bands (Fig. 2) and thus 2Δ(**k**). Changes of *q*_CDW_ alone can be ruled out to be driving an asymmetric closing of 2Δ since this would lead to a constant shift along the FS (see Supplementary Note 6 and Supplementary Fig. 5) and since time-resolved diffraction^29^ does not resolve such changes. In contrast, a transient modification of the electronic band dispersion, which can be parametrized with modified TB parameters, can explain the observed modification of the gap dispersion (Supplementary Fig. 5). The experimentally observed smaller gap dispersion can be reproduced by a reduction of *t*_⊥_ leading to a smaller curvature of the TB FS, illustrated in Figs 4e,f. This results in a slower deviation from perfect nesting of main and shadow bands along the FS, and hence an improved nesting, as shown by the increase of the grey area in Fig. 4f. In the limit of a vanishing curvature, the whole FS would be nested (we note that to maintain nesting for large changes of *t*_⊥_, also a slight change of *q*_CDW_ is required). To capture the experimental change in gap dispersion a reduction of *t*_⊥_ as large as 25% in the TB model is necessary (see Supplementary Note 7 and Supplementary Fig. 6). This scenario also explains the symmetric gap reduction at the point of perfect nesting ((*k*_*x*_, *k*_*z*_)∼(0.17, 0.29) Å^−1^) where 2Δ is symmetric to *E*_F_. We conclude to observe a trend towards enhanced nesting, which was not observed as a function of temperature^17^,^18^ and must be unique for non-equilibrium conditions.

The gap dynamics is linked to ion motion, as evidenced by the coherent oscillations of 2Δ. We speculate that such directed ion motion in the transient potential of the CDW distortion could transiently modify the orbital overlap in the network of Te orbitals and lead to the observed modification of the electronic dispersions and the improved FS nesting concluded above. As such, the enhanced nesting stabilizes the CDW in the optically excited state, however, in severe competition with fluctuations due to the excess energy, which destabilizes the CDW. The dynamics of the transient CDW state are therefore governed by the balance of these competing contributions. The limit in reduction of 2Δ≥0.17 eV observed in Fig. 3 originates from compensation between the increased fluctuations and the enhanced nesting, with their respective destabilizing and stabilizing effects on the CDW. While increasing fluctuations are the direct consequence of pumping at larger *F*, the stabilizing tendency is evidenced by the increasingly improved nesting for higher *F* (inset of Fig. 4d).

The possibility of modifying the transient electronic dispersion by optical excitation promises future control pathways for transient order in broken symmetry ground states of quantum materials as a complementary approach to nonlinear phononics^12^,^15^. Optimization of the excitation conditions may lead to desired ordered states, which requires modifications of the competing contributions towards stabilization and destabilization of the ordered state. Such a controlled approach might well be possible by adjusting pump photon energy and intensity since a reduction of photon energy results in lower excitation probability of secondary processes and related fluctuations while intensity defines the instantaneous excited state potential.

## Methods

### Static high-resolution ARPES measurements

Single crystals of RTe_3_ were grown by slow cooling of a binary melt^22^ and cleaved in ultrahigh vacuum (base pressure <7 × 10^−11^ mbar) at *T*=10 K. Static FS maps of HoTe_3_ (*T*_c1_=285 K, *T*_c2_=120 K) at *T*=180 K and *T*=300 K have been obtained using a laser-based ARPES setup with *hν*=7 eV and using a hemispherical electron analyzer (Scienta SES2000) (ref. 30). Energy and momentum resolution were better than 5 meV and 0.005 Å^−1^, respectively.

### Time-resolved ARPES measurements

For the trARPES experiments, the output of a commercial amplified Ti:sapphire laser system (Coherent RegA 9050) operating at 300 kHz repetition rate was used. Part of its output was frequency-quadrupled to yield *hν*=6.0 eV and used as probe pulses, while another part of the fundamental beam was time-delayed for excitation of the DyTe_3_ (*T*_c1_=305 K, *T*_c2_=50 K) sample, held at *T*=30 K during the measurements. The sample was mounted on a 45° slanted sample holder and oriented by Laue diffraction before trARPES measurements, which is necessary to reach the FS of RTe_3_ at a typical kinetic energy of 0.9 eV and large emission angles of >50°.

### Position-sensitive time-of-flight spectrometer

A self-built position-sensitive time-of-flight spectrometer^24^ was used for photoelectron detection, mounted inside an ultrahigh vacuum chamber with a base pressure of <7 × 10^−11^ mbar. It enables simultaneous access to both in-plane momentum components *k*_*x*_ and *k*_*z*_, along with the kinetic energy of the electrons, and thus allows efficient mapping of the electronic band structure. It consists of a field-free drift tube for photoelectrons, combined with a micro channel plate and two-dimensional delay line detector (RoentDek Hexanode Hex80). From the arrival time and impact position on the micro channel plate, the electron kinetic energy *E*_kin_ and both in-plane momentum components *k*_*x*_, *k*_*z*_ of each single photoelectron are calculated and stored in a three-dimensional (*E*, *k*_*x*_, *k*_*z*_) grid^24^. The Hexanode design of the detector allows for efficient detection of multiple electrons per laser pulse, which is a prerequisite for obtaining high-quality trARPES data as presented in this work at typical count rates of ∼150 kHz.

The overall temporal, spectral and momentum resolutions of the setup were 100 fs, 50 meV and 2 × 10^−3^ Å^−1^, respectively. For further details of the experimental setup see Schmitt *et al*.^6^,^23^.

## Additional information

**How to cite this article:** Rettig, L. *et al*. Persistent order due to transiently enhanced nesting in an electronically excited charge density wave. *Nat. Commun.* 7:10459 doi: 10.1038/ncomms10459 (2016).

## Supplementary Material

Supplementary Information Supplementary Figures 1-6, Supplementary Notes 1-7, and Supplementary References

Supplementary Movie 1 Movie of the transient Fermi surface. The red marker indicates the pump-probe delay.

Supplementary Movie 2 Movie of the transient electronic structure along k1, perpendicular to the Fermi surface. The red marker indicates the pump-probe delay

Supplementary Movie 3 Movie of the transient electronic structure along k2, along the Fermi surface. The red marker indicates the pump-probe delay.
