# Supplementary material for: Persistent order due to transiently enhanced nesting in an electronically excited charge density wave
Source: Nat Commun. 2016 Jan 25;7:10459. doi: 10.1038/ncomms10459 (PMC4737756; doi:10.1038/ncomms10459)
Supplement: Supplementary Information — Supplementary Figures 1-6, Supplementary Notes 1-7, and Supplementary References [file ncomms10459-s1.pdf]

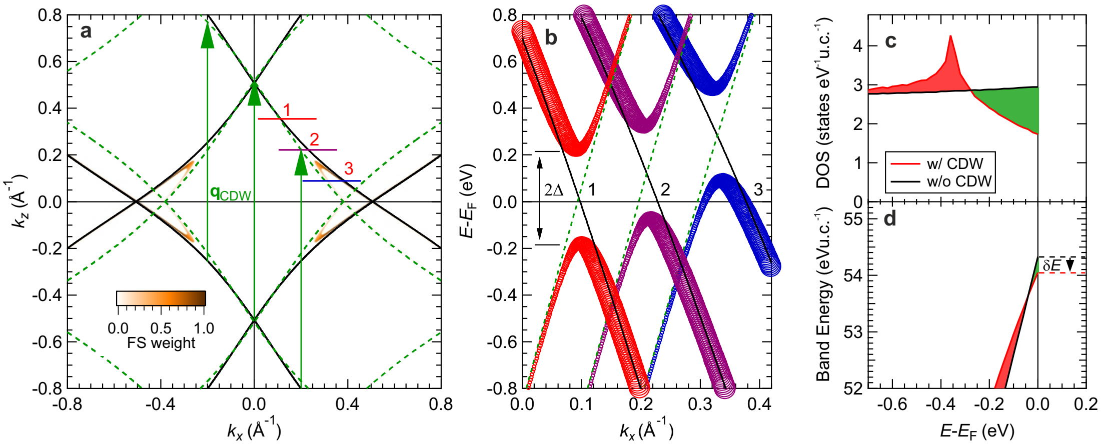

Supplementary Figure 1: Tight binding model of RTe_3. (a) Fermi surface weight of the interacting TB model on a color scale. Black lines are main TB bands, and green dashed lines are shadow bands translated by $\pm\mathbf{q}_{\mathrm{CDW}}$, shown by the green arrows. Lines 1-3 mark the positions of the dispersions shown in (b). (b) Band dispersions along lines (1-3) shown in (a) for $\Delta = 0.2\,\mathrm{eV}$. Black lines are main TB bands, green dashed lines shadow bands, and colored markers are the dispersion of the interacting TB model, where the size of the markers in proportional to the spectral weight. Note the shift of the gap 2Δ together with the crossing of main and shadow bands. (c) Density of states (DOS) of non-interacting (black) and interacting (red) TB model. Red and green areas denote the shift of states due to the CDW gap. (d) Electronic energy for non-interacting (black) and interacting (red) TB model. The energy gain due to the CDW gap δE is indicated.

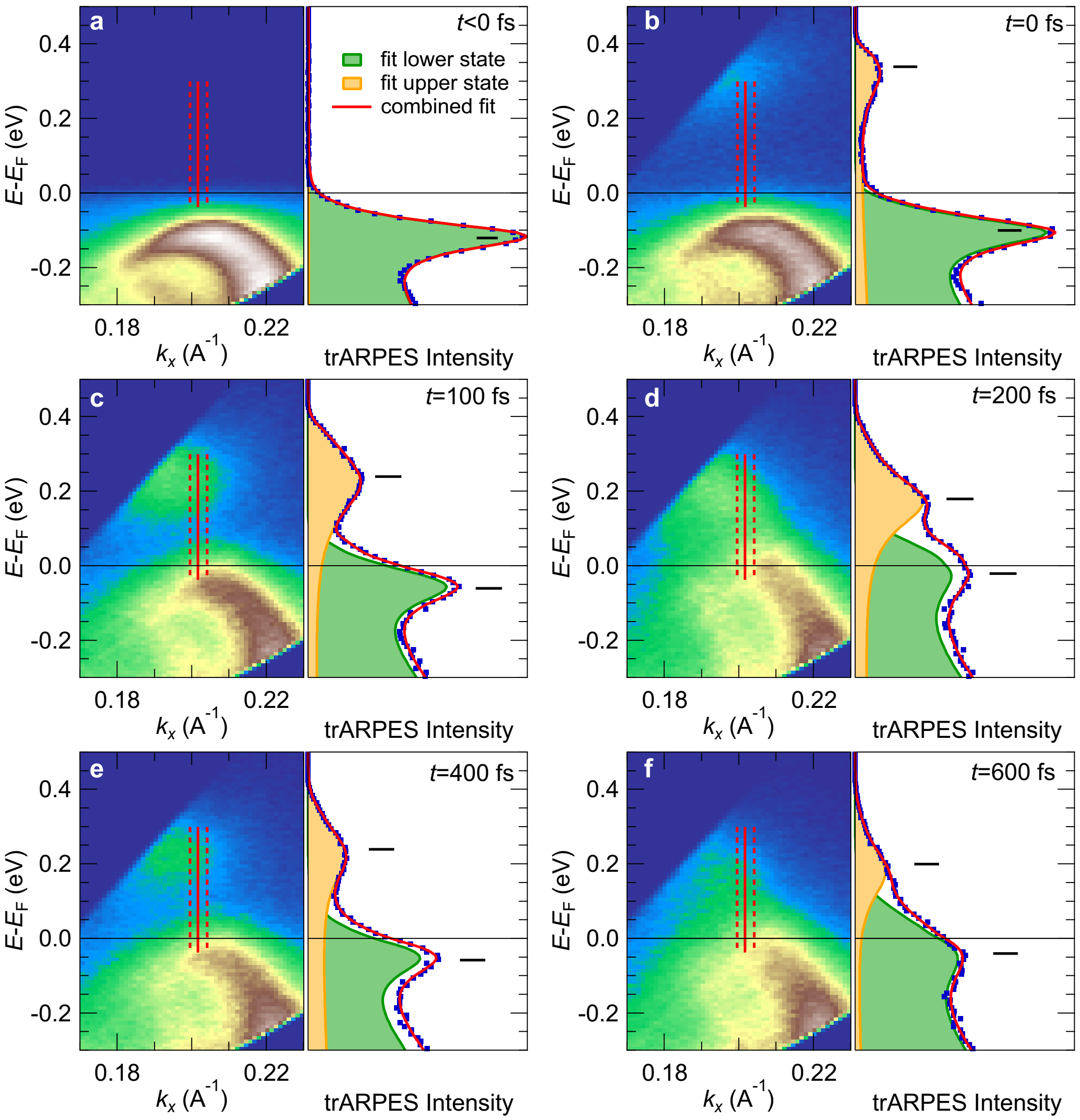

Supplementary Figure 2: Fitting of the transient CDW peak positions. (a)-(f) Each panel shows on the right the transient trARPES intensity (blue dots) integrated between the dashed lines in the left dispersion graph (see Fig. 2 of the main text). Fitting function of occupied states (green), unoccupied states (yellow) and the total fitting function (red) are shown (see text). Black marks indicate extrema of the second derivative with respect to energy of the data.

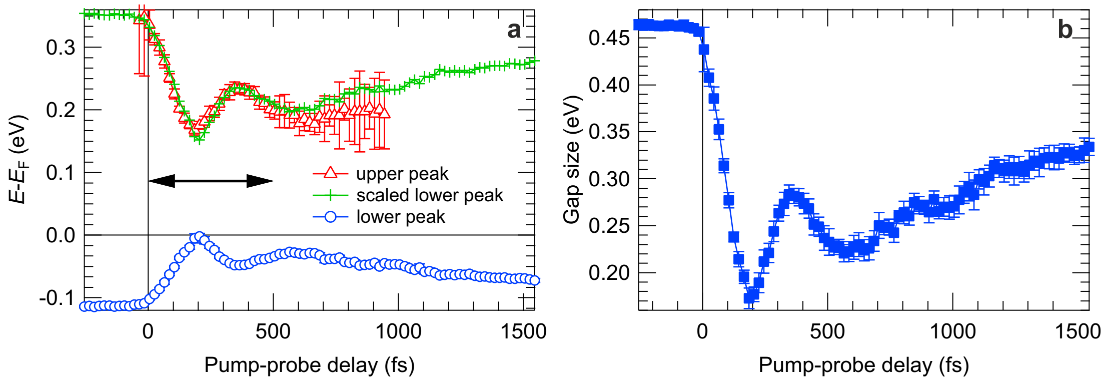

Supplementary Figure 3: Determination of the transient CDW gap size. (a) Lower peak position (blue) and upper peak position (red) compared to the lower peak position, inverted and scaled to the upper peak position between 0 fs and 500 fs (green). Error bars are 95% confidence intervals of the fitted peak positions. (b) CDW gap size determined from the difference of lower and upper CDW peak position. For $t < 0$ fs and $t > 500$ fs, the scaled lower peak position has been used for the upper peak position. Error bars are derived from 95% confidence intervals of fitted peak positions.

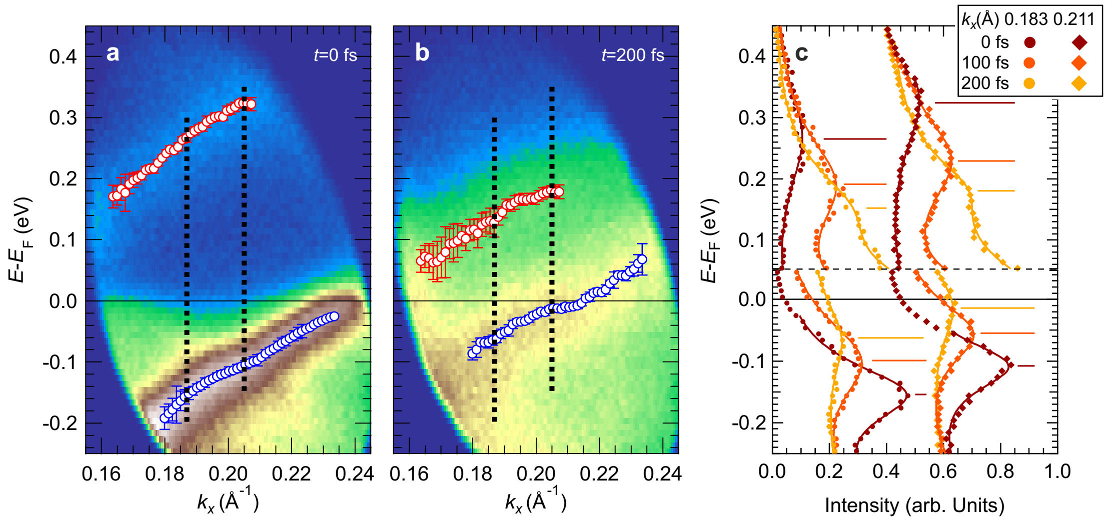

Supplementary Figure 4: Fitting of trARPES spectra along the Fermi surface. (a, b) trARPES spectra along k_2 (see main text) parallel to the Fermi surface, for $t = 0$ fs and $t = 200$ fs. The momentum position is indexed by k_x for simplicity here. Markers are peak positions determined by Lorentzian line fits, and dashed lines show the positions of the spectra shown in (c). Error bars are 95% confidence intervals of the fitted peak positions. (c) trARPES spectra at various pump-probe delays, at momentum positions indicated in panels (a-b). Spectra at $E > E_F$ are multiplied by a factor of 2.5, and spectra at $k_x = 0.221\,\text{Å}^{-1}$ are offset for clarity. Lines are Lorentzian fits to the data, and horizontal lines indicate the peak positions determined by the fits.

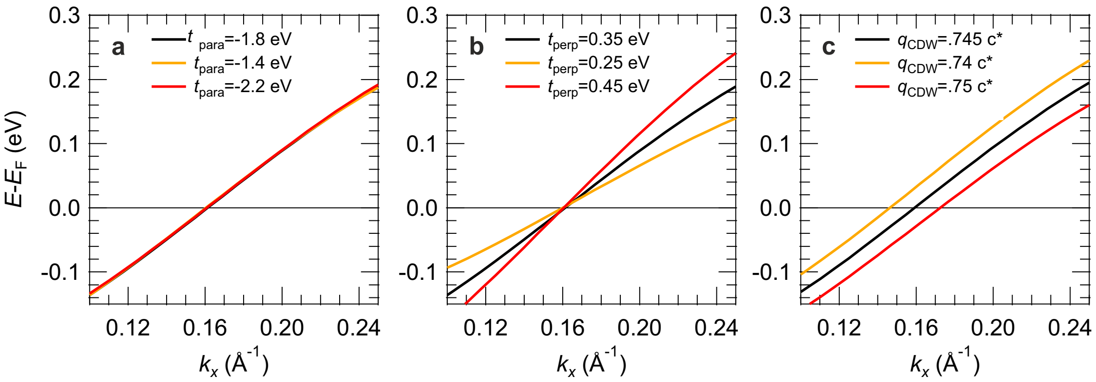

Supplementary Figure 5: Position of the gap center in the tight binding model. Position of the gap center (crossing of main and shadow band) in the tight binding model for a variation of (a) $t_{\parallel}$, (b) $t_{\perp}$ and (c) q_{CDW} as a function of k_x along the Fermi surface. While a change of q_{CDW} mainly shifts the whole gap in energy, a change in $t_{\perp}$ reproduces the change in slope observed in the transient state.

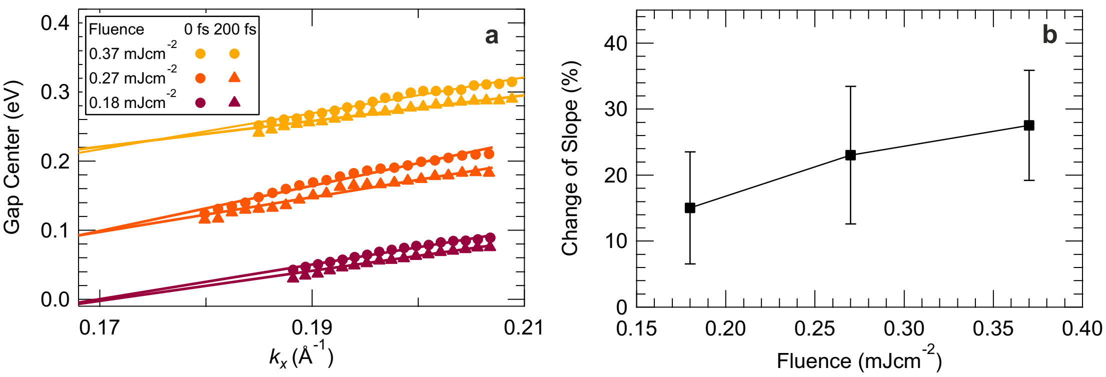

Supplementary Figure 6: Fluence dependent shift of the gap center. (a) Position of the gap center as a function of momentum along k_2, for $t = 0\,\text{fs}$ (circles) and $t = 200\,\text{fs}$ (triangles) for various fluences. Pairs of traces are vertically offset for clarity. Lines are linear fits. (b) Relative change of the slope of the gap center extracted from the fits in (a), as a function of absorbed fluence. Error bars denote 95% confidence intervals, and lines are guides to the eye.

Supplementary Note 1

Tight Binding model of RTe_3

The quasi two-dimensional electronic band structure close to the Fermi level in RTe_3 can be well approximated by a simplified tight binding (TB) model of the in-plane Te $5p_x/z$ orbitals, that exhibit coupling along and perpendicular to the chains, $t_\|$ and $t_\perp$, see Fig. 1a of the main manuscript. This leads to the following band dispersion [1]:

$$E_{p_x}(k_x, k_z) = -2t_\| \cos([k_x + k_z] \cdot a/2) - 2t_\perp \cos([k_x - k_z] \cdot a/2) - E_\mathrm{F} \quad , \tag{1}$$

$$E_{p_z}(k_x, k_z) = -2t_\| \cos([k_x - k_z] \cdot a/2) - 2t_\perp \cos([k_x + k_z] \cdot a/2) - E_\mathrm{F} \quad , \tag{2}$$

with $E_\mathrm{F} = -2t_\| \sin(\pi/8)$ fixed by the band filling, and the TB coupling parameters $t_\| \approx -1.9\,\mathrm{eV}$ and $t_\perp \approx 0.35\,\mathrm{eV}$ chosen to match the experimental Fermi surface. The bands of this non-interacting TB model, shown in Supplementary Fig. 1(a) as black solid lines, gain a small curvature proportional to the ratio $t_\perp/t_\|$ leading to the diamond-shaped Fermi surface (compare to Fig. 1 of the main manuscript).

Nesting of this metallic FS along the nesting vector $\mathbf{q}_\mathrm{CDW}$ along c^* transfers spectral weight into shadow bands translated by $\pm\mathbf{q}_\mathrm{CDW}$ (green dashed lines). The coupling between bare bands $|\mathbf{k}\rangle$ and shadow bands $|\mathbf{k} \pm \mathbf{q}\rangle$ of strength Δ is taken into account by considering the following wave function which allows for the coupling of states $|\mathbf{k}\rangle$ and $|\mathbf{k} \pm \mathbf{q}\rangle$, which was termed interacting TB model in the literature [2, 1, 3]:

$$|\Psi_k\rangle = u_{k-q}\,|\mathbf{k} - \mathbf{q}\rangle + u_k\,|\mathbf{k}\rangle + u_{k+q}\,|\mathbf{k} + \mathbf{q}\rangle \quad , \tag{3}$$

where the coefficients are determined as the solutions of the coupling matrix:

$$M = \begin{pmatrix} \epsilon_{p_{x/z}}(\mathbf{k} - \mathbf{q}) & \Delta & 0 \\ \Delta & \epsilon_{p_{x/z}}(\mathbf{k}) & \Delta \\ 0 & \Delta & \epsilon_{p_{x/z}}(\mathbf{k} + \mathbf{q}) \end{pmatrix} \quad . \tag{4}$$

This leads to the opening of gaps 2Δ around the crossing of main and shadow bands, and splitting of the bands into two branches [4]:

$$E_{1/2}(\mathbf{k}) = \frac{\epsilon(\mathbf{k}) + \epsilon(\mathbf{k} \pm \mathbf{q})}{2} \pm \sqrt{\left(\frac{\epsilon(\mathbf{k}) - \epsilon(\mathbf{k} \pm \mathbf{q})}{2}\right)^2 + \Delta^2} \quad , \tag{5}$$

see Supplementary Fig. 1(b). Due to the slight mismatch in curvature of main and shadow band, the band crossing and hence the position of the gap relative to E_F shifts with momentum, leading to residual metallic pockets on the FS where the lower band shifts above E_F, see FS in Supplementary Fig. 1(a) and blue curve in Supplementary Fig. 1(b). To model the data in the main manuscript we included in addition a coupling of p_x and p_z bands and 3-dimensional folding of the bands [1, 3], leading to a slight shift of the gap position with respect to the band crossing.

The gain in electronic energy due to the opening of the gaps in the electron dispersion can be estimated from the density of states (DOS) $\rho(\epsilon)$. Supplementary Fig. 1(c) shows the DOS of interacting and non-interacting TB model, as red and black lines, respectively. The opening of the gap leads to a transfer of states from the Fermi level to lower energies, shown by red (increase) and green (decrease) colors. The electronic energy in the valence band is given by

$$E = \int_0^{\epsilon_\mathrm{F}} \rho(\epsilon) \cdot \epsilon \, \mathrm{d}\epsilon, \tag{6}$$

where $\epsilon_F \approx 6$ eV from the band bottom. Supplementary Fig. 1(d) shows the electronic energy for the interacting and non-interacting TB model, yielding a gain in electronic energy $\delta E = E_{\text{non-interacting}} - E_{\text{interacting}} \approx 250\,\text{meVu.c.}^{-1}$. For these estimates, q_{CDW} has been adjusted accordingly to keep the integrated DOS at E_F constant.

Supplementary Note 2

Determination of the transient CDW band positions

For the determination of the position of the CDW bands, transient trARPES spectra are fitted, as shown for exemplary pump-probe delays in Supplementary Fig. 2. To account for the highly non-thermal distribution of electrons within the first picosecond, a fitting model composed of two independent parts for the occupied and unoccupied band structure was considered, shown as green and yellow areas, respectively. Each part consists of a linear background and a Lorentzian peak function, multiplied with a Fermi-Dirac distribution:

$$I(E,t) = \sum_{i=1}^{2} \left[A_i(t) + B_i(t) \cdot E + \frac{C_i(t)}{(E - E_i(t))^2 + W_i(t)^2} \right] \left(\exp\left[\frac{E - \mu_i(t)}{k_B T_i(t)} \right] + 1 \right)^{-1} \quad . \quad (7)$$

Here, $i = 1, 2$ are the fit functions of occupied and unoccupied bands, A_i and B_i the coefficients of a constant and linear backgrounds, C_i, E_i and W_i the amplitudes, positions and widths of the Lorentzians, and μ_i and T_i the chemical potential and electronic Temperature of the Fermi-Dirac distributions. The position of the Fermi-Dirac distribution of the unoccupied bands was used as an auxiliary parameter to mimic the cut-off at high energies due to the dispersion of the acceptance angle of the pTOF (see the image plots in Supplementary Fig. 2). We find an excellent description of the data by the fits for all pump-probe delays. Due to the highly non-thermal electronic system after excitation, even the Fermi-Dirac distribution used for the formally occupied part of the spectrum needs to be considered as an auxiliary parameter, as no Fermi level can be defined any more in the spectrum e.g. at $t = 200$ fs, and is held fixed with a high value of T_1 to produce a smooth curve. In order to crosscheck the results of the fitting procedure, peak positions are compared to the extrema in the second derivative of the data with respect to energy, as commonly used in ARPES [5], confirming the results of the fitting procedure (black markers in Supplementary Fig. 2).

Supplementary Note 3

Determination of the CDW gap size

Due to the progressing thermalization of the electronic system, the position of the upper CDW band becomes hard to determine on top of the background of the hot thermalized electron distribution for $t > 500$ fs and is indistinguishable from the background for delays $t > 1000$ fs. In order to determine the transient gap size for all pump-probe delays, we compare the occupied band position inverted and scaled by a factor of $\times 1.8$, shown as green markers in Supplementary Fig. 3(a), to the unoccupied band position. We find a close agreement for $0\,\text{fs} < t < 500$ fs (black arrow in Supplementary Fig. 3), indicating a constant asymmetry of the gap closing with pump-probe delay. The deviations towards larger band shift of the unoccupied band found for $t > 500$ fs correspond to a systematic underestimation of the peak energy, which can be explained by the asymmetric peak shape due to the exponential background of the hot electron distribution and manifests in the large error bars (see fits in Supplementary Fig. 2). Thus, for delays at $t < 0$ fs

and $t > 500\,\text{fs}$, where no reliable determination of the unoccupied peak position is possible, we extrapolate the unoccupied peak position by the scaled occupied peak position, assuming a constant proportion of occupied and unoccupied peak shift. This procedure yields the transient CDW gap size, shown in Supplementary Fig. 3(b) and Fig. 3 of the main text.

Supplementary Note 4

Determination of the pump energy density

The absorbed pump fluence per pulse F_{abs} in the limit of a Gaussian pump beam profile much larger than the probe beam is given by [4]:

$$F_{\text{abs}} = E_{h\nu} \cdot (1 - R) \cdot \frac{4\ln(2)}{\pi} \cdot \frac{\cos(\alpha)}{\text{FWHM}_x \cdot \text{FWHM}_y} \quad , \tag{8}$$

where $E_{h\nu}$ is the pump energy per pulse, R the reflectivity of the sample, α the angle of incidence with respect to the surface normal, and $\text{FWHM}_{x/y}$ the full-width at half maximum of the horizontal and vertical pump profiles. The absorbed pump energy density per unit cell $\epsilon_{\text{uc}}(z)$ as a function of depth z from the surface decays with the optical penetration depth $d_{h\nu} \approx 24\,\text{nm}$ as

$$\epsilon_{\text{uc}}(z) = \epsilon_{\text{1st}} \cdot \exp(-z/d_{h\nu}) \quad , \tag{9}$$

where ϵ_{1st} is the energy density in the 1st unit cell with volume $V_{\text{uc}} \approx 480\,\text{Å}^3$ [6] at the surface

$$\epsilon_{\text{1st}} = F_{\text{abs}} \cdot V_{\text{uc}}/d_{h\nu} \quad . \tag{10}$$

For a photoemission probe depth d_{probe} much smaller than $d_{h\nu}$, the effective pump energy density is just given by ϵ_{1st}.

Supplementary Note 5

Transient CDW band dispersion parallel to the Fermi surface

The electronic band dispersion along k_2 parallel to the Fermi surface (see Fig. 1c and Fig. 4 of the main manuscript) were determined separately for occupied and unoccupied states, as a function of momentum. Peak positions were determined by fits of a Lorentzian line shape, plus an exponential background function:

$$I(E,t) = A(t) + B(t) \cdot \exp(-\frac{E}{\epsilon(t)}) + \frac{C(t)}{(E - E_0(t))^2 + W(t)^2} \quad . \tag{11}$$

Here, A is a constant offset, B and ϵ the amplitude and characteristic energy of the exponential background, and C, E_0 and W the amplitude, position and width of the Lorentzian. Solid lines in Supplementary Fig. 4(c) show the fits to the data for two momentum positions, and for selected pump-probe delays. For these fits, no Fermi function was considered, and fits restricted in energy and momentum accordingly. The transient change of the gap dispersion discussed in the main manuscript manifests in the slightly larger shift with pump probe delay of the occupied peak at $k_x = 0.183\,\text{Å}^{-1}$, and of the unoccupied peak at $k_x = 0.211\,\text{Å}^{-1}$

Supplementary Note 6

Position of the gap center in the tight binding model

The energetic position of the CDW gap in the band structure is determined in the tight binding model by the crossing of the main p_x/p_z bands and the CDW shadow bands that are translated by $\pm\mathbf{q}_{\mathrm{CDW}}$ (compare Supplementary Fig. 1(b)). In order to estimate the effect of the various parameters of the TB model, the position of this crossing of main and shadow band in the non-interacting TB model has been determined for a variation of $t_\perp$, $t_\parallel$ and q_{CDW}, shown in Supplementary Fig. 5(a-c) as a function of k_x coordinate, respectively[1]. While we find virtually no change in the gap position upon changing $t_\parallel$, a change of $t_\perp$ nicely reproduces the change in slope of the gap dispersion observed in the transient excited state (see Supplementary Fig. 6 and Fig. 4 of the main text). We find the slope to be proportional to $t_\perp$ over a large range of values. In contrast, a change in q_{CDW} mostly leads to a shift of the whole gap dispersion, while affecting the slope only very little (Supplementary Fig. 5(c)).

Supplementary Note 7

Fluence dependent shift of the gap center

In order to investigate the fluence dependence of the transient change of the gap dispersion discussed in the main manuscript, the gap position along the Fermi surface (direction k_2 in Fig. 1 (c) of the main manuscript) has been determined from peak fits to the upper and lower CDW peak for various fluences. Supplementary Fig. 6(a) shows the gap center for various fluences as a function of momentum along the Fermi surface at $t = 0\,\mathrm{fs}$ (circles) and $t = 200\,\mathrm{fs}$ (triangles), where the gap is minimal. For all fluences, we observe a momentum-dependent shift of the gap center towards the Fermi level during the collapse of the gap, corresponding to a change of the band structure towards a more square-like Fermi surface. In order to quantify this change of the band structure, the slope of the gap center along the Fermi surface has been determined by linear fits (lines in Supplementary Fig. 6(a)) and the relative change of the gap dispersion is shown in Supplementary Fig. 6(b) as a function of absorbed fluence (also shown in the inset of Fig. 4(d) of the main manuscript). Despite the large error bars, the change in slope is distinctly different from zero for all fluences and a clear trend towards larger change in the band dispersion for higher excitation is observed.

Supplementary References

[1] Brouet, V. *et al.* Angle-resolved photoemission study of the evolution of band structure and charge density wave properties in RTe_3 (R=Y, La, Ce, Sm, Gd, Tb, and Dy). *Phys. Rev. B* **77**, 235104 (2008).

[2] Voit, J. *et al.* Electronic Structure of Solids with Competing Periodic Potentials. *Science* **290**, 501–503 (2000).

[1] Note that in order to reproduce the symmetric gap around E_{F} at $k_x \sim 0.17\text{Å}^{-1}$ in this simplified model, a slightly larger value of $q_{\mathrm{CDW}} \approx 0.745c^*$ than observed experimentally [6] has been used. In the interacting TB model, the interaction between p_x and p_z leads to an additional shift of the CDW gap to lower energies close to the corner of the diamond shaped Fermi surface, which reproduces the correct gap position for values of q_{CDW} in agreement with the experimental values [3].

[3] Moore, R. G. *et al.* Fermi surface evolution across multiple charge density wave transitions in $ErTe_3$. *Phys. Rev. B* **81**, 073102 (2010).

[4] Leuenberger, D. *et al.* Classification of collective modes in a charge density wave by momentum-dependent modulation of the electronic band structure. *Phys. Rev. B* **91**, 201106 (2015).

[5] Damascelli, A., Hussain, Z. & Shen, Z.-X. Angle-Resolved Photoemission Studies of the Cuprate Superconductors. *Rev. Mod. Phys.* **75**, 473 (2003).

[6] Ru, N. *et al.* Effect of chemical pressure on the charge density wave transition in rare-earth tritellurides RTe_3. *Phys. Rev. B* **77**, 035114 (2008).
